# Suicidal Behaviour as an Emerging Factor in Female Victims of Gender-Based Violence within a Relationship: An Exploratory Study

**DOI:** 10.3390/ijerph192215340

**Published:** 2022-11-20

**Authors:** Ismael Puig-Amores, Isabel Cuadrado-Gordillo, Guadalupe Martín-Mora-Parra

**Affiliations:** Department of Psychology and Anthropology, University of Extremadura, 06071 Badajoz, Spain

**Keywords:** suicidal behaviours, intimate partner violence, risk factors

## Abstract

Intimate partner violence (IPV), in addition to being an important public health problem, is a risk factor for suicidal behaviour (SB). The objective of this study was to explore the risk of suicidal behaviour associated with the different forms of abuse and the consequences derived from it. This exploratory study was conducted on a sample of women who experienced IPV (*N* = 70) in the Extremadura Region (Spain). Demographic factors, abuse experience, suicidal ideation (SI), suicidal communication (SC), and suicide attempts (SAs) were analysed according to the type of abuse. We found that a very high prevalence of psychological violence (IPVp) was accompanied, in most cases, by some other type of abuse (IPVp+). Additionally, a significant relationship was found between IPV and SB. Logistic regression revealed a greater risk of SI and SA in the IPVp+ group. The results of this study could be useful to healthcare service professionals in preventing suicidal behaviour.

## 1. Introduction

Physical, sexual, or psychological violence by a partner or ex-partner (IPV) is the commonest form of violence against women [1]. Such is its impact that it is considered to be a serious public health and human rights problem [2]. According to the most recent meta-analysis [1], between the years 2000 and 2018, the prevalence of this type of violence was 27%, so it is estimated that up to 753 million women who were married or with partners over 15 years of age have undergone IPV at least once in their lives. In the region of Extremadura (Spain), the scenery is equally alarming. According to the Macro-survey of violence against women of 2019, the prevalence of IPV+ in general population reaches 24% [3]. The seriousness of this problem has generated a continuous interest in factors related to IPV in order to develop explanatory models of this phenomenon and apply them in research [4,5,6]. Many factors of IPV have been extensively researched, including demographic characteristics such as age [7,8,9], marital status [8,9,10], level of education [9,10,11], employment status [12], relationship time with the aggressive partner [4], and alcohol or substance abuse [13,14,15,16]. Another factor of IPV is the abuse experienced, especially the different types women experience [17,18], and also a family history of abuse (childhood maltreatment, sexual abuse history, etc.) [5,7,9,19].

The abuse experienced can be very diverse [18]. Several studies indicate that psychological violence is usually present in most cases, either alone (IPVp) or combined with physical and/or sexual violence (IPVp+), being the most frequent form of abuse and one of the main causes of health problems in women [8,10,20,21,22]. IPV can have physical consequences [1,23], with the number of health problems being greater when the abuse has been temporally close, even lasting over time once the experience of abuse has ended [24]. In this sense, physical consequences such as chronic pain can generate depressive symptoms and hopelessness, two factors associated with an increased risk of suicidal behaviour [1,25,26,27,28,29]. In addition, IPV can cause neuropsychological [25,26] or psychological problems [27,28,29], including suicide attempts and suicidal ideation [15,30,31]. In Extremadura, a region of Spain, the main consequences of IPV were physical damage such as injuries, bruises, etc. (20–40%), depression (45.4%), anxiety (47.3%), sleeping or eating problems (48.6%), loss of self-esteem (61%), hopelessness (50%), and suicidal thought or suicide attempts (12.3%) [3].

Although these consequences can be associated with an increased risk of suicide [15,31,32], it is psychological abuse which has received the most attention in studies of suicidal behaviour. In this regard, hopelessness is especially relevant as it is key to the emergence of suicidal ideation (SI) [33,34,35] and suicide attempts (SAs) [35,36,37]. In this sense, it should be noted that depressive symptoms, including hopelessness, have a high incidence among female victims of IPV [15,24,38] and that hopelessness indeed acts as a mediator between the abuse and suicidal behaviour (SB) [39,40]. Likewise, a greater degree of association has been found between hopelessness and SAs when there is in fact a history of SAs [34,35,36,37,38]. Previous attempts can be considered to be a powerful predictor of subsequent attempts because they substantially increase the risk after a first SA [35,41,42,43]. Thus, hopelessness, SI, and SAs are among the main factors associated with the increased risk of SB [34,36,37,39,40,41,42,43], and consequently these three components are key for the evaluation of suicide risk in females exposed to IPV.

The difference between SI and SAs is another important factor to consider in the analysis of SB. These behaviours (SI and SAs) are currently considered to be closely related, although they require different explanations and have different predictors [34]. This fact is reflected in the “three-step theory of suicide” and “the integrated motivational-volitional model of suicidal behaviour” explained by authors such as [33,34,39,40]. These two theoretical framework try to explain the appearance of SI and its genesis until reaching SAs. In short, both postulate that the perception of the immutability of life circumstances (i.e., hopelessness), would play a key modulating role in the evolution and severity of the different types of SB, having been suggested that the emotional pain produced by any life event or personal circumstance could be the close antecedent of SI. Additionally, these explanatory frameworks of suicidal behaviour suggest, with greater or lesser emphasis, that the loss of fear of pain and death necessary to be able to carry out a suicidal act can come from genetic vulnerability, although it can also be acquired by habituation to physical and/or emotional pain after prolonged or chronic exposure to such stressors.

In the context of IPV, the perception of immutability (hopelessness) of the abuse experienced by women is considered to be the personal event or circumstance capable of triggering SI. In addition, the duration of the relationship, and the cohabitation period with the aggressor, would lead to a prolonged exposure to the abuses which, later, would increase the habituation to physical and/or emotional abuse. These two factors (hopelessness and habituation) are the key for losing the fear of pain and death, which could, ultimately, lead female victims of IPV to carry out SAs. From this perspective, the main goal of the present work was to explore the possible relationships between the consequences of abuse and SB in a group of female victims of IPV. Specifically, it is proposed that the physical consequences derived from the abuse (major health problems such as chronic pain syndromes, mobility limitations, and general poor health), the psychological consequences (especially hopelessness) deriving from IPV, or the isolation that these women usually experience in this type of abusive relationships could explain the emergence of suicidal behaviour.

### Research Hypotheses and Objectives

To this end, the first objective was to explore the prevalence of abuse experienced and the consequences of the abuse. The second objective was to investigate whether there is any association between demographic factors, the abuse experienced, and the consequences of the abuse with some form of IPV (IPVp or IPVp+). Finally, the third objective was to determine if there is an association connecting SI and SAs with demographic factors, the abuse experienced, and especially the consequences of the abuse. Based on these objectives, the following working hypotheses were posited:

(1) Psychological violence will be present in most cases and will occur to a greater extent combined with physical and/or sexual violence (IPVp+) than alone (IPVp).
(2) IPVp+ will be significantly related to both SI and SAs, additionally presenting a greater risk of developing SB than will IPVp.
(3) The hopelessness variable will be significantly related to IPV and SB, with hopelessness implying a greater risk of SI and SAs.

## 2. Materials and Methods

### 2.1. Participants

The sample (*N* = 70) was made up of a group of women in psychological therapeutic process at 6 Psychological Care Points (PAPs) of the Care Network for Victims of Gender Violence in Extremadura (Spain). The only exclusion criterion was being under 18 years of age. The age of the participants ranged from 19 to 62 years old (M = 40.14; SD = 10.71).

### 2.2. Procedure

Prior to beginning the study, authorization was obtained from the General Director of the Women’s Institute of Extremadura, as well as from the Ministry of Equality & Cooperation for Development of the Junta de Extremadura. Subsequently, authorization for the interviews was requested from the management of each of the PAPs. After permits were obtained, the PAPs were contacted by telephone. In this first contact, the objectives of the study, the content of the interviews, and their approximate duration were explained. The anonymity of all the participants was also guaranteed. The interviews began in 2020 and continued until March 2021. The interviews were carried out by the researchers involved in this study. The participants completed the questionnaires in a calm and relaxed environment, with enough time to discuss and comment on the questions. Before completing the questionnaires, the explicit informed consent of the participants was requested, and information was included regarding the use of the data, the objective of the study, and the voluntary participation and assurance of confidentiality and anonymity. The acquisition and processing of data was carried out according to the Spanish Organic Law 3/2018 of December 5 on the Protection of Personal Data and the Guarantee of Digital Rights.

### 2.3. Questionnaires and Variables

Semi-structured interview with victims of domestic abuse were conducted [44]. This questionnaire comprises 21 items, grouped into two blocks. The first of these sections (items 1 to 10) used a polytomous scale that compiled demographic information such as age, marital status, and profession. The second block had a dichotomous scale. This second section was subdivided into three: the first one (items 11–14) was designed to measure the characteristics of the abusive relationship, the second (item 20), the antecedents and consequences of psychological abuse, and the last (item 21) explored the history of psychological treatment. Specifically, using this instrument, the demographic factors of the victims (age, cohabitation, educational level, employment status), the type of abuse (IPVp or IPVp+), and the abuse experienced (years of cohabitation and relationship with the aggressive partner, family history of abuse, danger to life, running away from home, lawsuits filed, and psychological treatment before and after the abuse) were registered.

To measure the variables related to SB, the Spanish validation of the Plutchik Suicide Risk Scale was used [45]. This questionnaire comprises 15 items with a dichotomous scale (yes/no). Each affirmative answer is scored with 1 point, and the negative answers score 0 points. The total is the sum of the scores of all items. Using this scale, the risk of suicide present in the participants was obtained (cut-off point = 6). On the other hand, by analysing the answers given to items 13, 14, 15, and 11 the presence of SI, SC, SAs, and the family history of suicide were obtained. Additionally, the analysis of this scale allowed us to determine the presence of hopelessness, depressive symptomatology (feeling depressed, pessimistic future, feelings of failure, little interest in relating to people, feelings of anger towards others), sleep problems, and sleep medication. This instrument shows a sensitivity and specificity of 88% to detect people in risk of committing suicide, and Cronbach’s alpha 0.90 for the Spanish population.

### 2.4. Statistical Analysis

The statistical analysis was performed using the Statistical Package for the Social Sciences (SPSS v.26; IBM Corp. Armonk, NY, USA). The analysis began with descriptive statistics on demographic variable. Secondly, to analyse the relationships between the variables of the study, intergroup analyses were performed (SI vs. no SI; presence of SAs vs. absence of SAs; and psychological IPV vs. psychological IPV in combination with other types of violence). Analyses of dichotomized variables were performed using Bernoulli’s test, Pearson’s χ^2^ test, and Fisher’s exact test. Meanwhile, the variables age, years of relationship with the partner, years of cohabitation with the partner, and the risk of suicide were analysed using the Mann–Whitney U statistical test. Finally, logistic regressions were performed using the (conditional) successive backward stepwise method with the set of variables that were statistically significant in the intergroup analyses. Odds ratios (OR) were reported for this statistical test.

## 3. Results

### 3.1. Sample Description

The results revealed that 64.3% of the victimized women were between 30 and 49 years old, 41.4% were single, and 47.1% were separated. Most of the women (70%) lived with their own family (with their children), in an active employment status in some cases and unemployed in others. It was observed that 51.4% had secondary education (Table 1). On the other hand, it should be noted that the other age groups occurred in proportions of less than 20%, both women of under 29 years of age and women over 50 years of age. In the same sense, only 11.4% of the women were married, and very few lived alone (10%) or with their family of origin (20%).

### 3.2. The Association between Suicidal Behaviors and Demographic Factors

The first analysis showed a statistically significant relationship between the variable employment status and the variable suicidal ideation (χ^2^ = 4.509; *p* < 0.005). The data showed that, in the group of women with SI, being unemployed was more frequent than being employed (31.8%), while in the group of women without SI, this proportion is reversed, with it being more frequent for them to be in an actively employed status (57.7%) than unemployed (38.5%). Likewise, the variable “years of cohabitation with the partner” presents a statistically significant relationship with SI (χ^2^ = 5.544; *p* < 0.005). The results showed a lower frequency of SI among women who had cohabitated with the aggressor for less than a year, while most women who had SI had cohabitated with their aggressor for longer than 10 years.

Additionally, the intergroup analysis showed that marital status had a statistically significant relationship with SAs (χ^2^ = 4.301; *p* < 0.005). This fact seems to indicate a greater trend of SAs among separated or divorced women. In this sense, it is observed that, in the group of women who had attempted suicide, 62.5% were separated or divorced, while in the group of women who did not have SAs, the frequency of separated or divorced women was 39.1% (Table 1).

### 3.3. Background, Abuse Experienced, Types of Violence Suffered, and Consequences of Victimization

The second analysis indicated a statistically significant relationship between the existence of a history of abuse within the family and violence in intimate relationships, with this association being more frequent when the women had suffered IPVp+ (χ^2^ = 4.624; *p* < 0.005). In parallel, it was found that a third of the victimized women had received psychological treatment prior to experiencing abuse in a partner relationship (*p* < 0.005). In these cases, a statistically significant relationship was revealed between having received psychological treatment before the abuse experienced and suffering SI (*p* < 0.005). It was also detected that substance abuse is rare in this group of victimized women (2.9%; *p <* 0.001) (Table 2).

Regarding the abuse experienced, the analysis showed that psychological violence was experienced in 100% of the cases, either alone (31.4%), or combined with physical or sexual violence (68.6%), the latter being the more frequent (*p <* 0.005). On the other hand, contrary to what was expected, it was found that only a third of the women suffered from some important health problem (*p <* 0.005). Additionally, it was observed that, during the period of time the abuse lasted, 85.7% of the women felt that their lives were in danger, resulting in 64.3% of women being forced to flee their home to protect their lives, and later filing complaints regarding their abuser in 72.9% of the cases (Table 2). Likewise, it was found that the group of women who suffered IPVp+ felt danger to their own lives to a greater extent than the rest of the victimized women (93.8%; χ^2^ = 8.054; *p <* 0.005).

Finally, focusing on the consequences of the abuse experienced, the analysis further indicated that 85.7% of the women needed psychological treatment after the abuse and that 80% had sleep problems, although almost half revealed they had no need for medication to sleep. Most of the victimized women experienced negative emotions related to feelings of worthlessness (91%) and wanting to abandon everything (90%). Nonetheless, only 47.1% reported feeling depressed. Likewise, contrary to expectations, hopelessness turned out to be an infrequent consequence of abuse in the sample (25.7%) (Table 3).

The intergroup analyses showed statistically significant associations between SAs and major health problems, feelings of anger towards others, and having little interest in relating to people (*p* < 0.005). Likewise, a statistically significant relationship was found between SI and having little interest in relating to people (*p* < 0.005). Additionally, statistically significant associations were observed between hopelessness and the two suicidal behaviours (SI and SAs), but not with the type of violence. Another relevant finding showed a significant association between SC and SAs (*p* < 0.005). Similarly, there was an even stronger association between SC and SI (*p* < 0.001) (Table 3).

**Figure 1:**
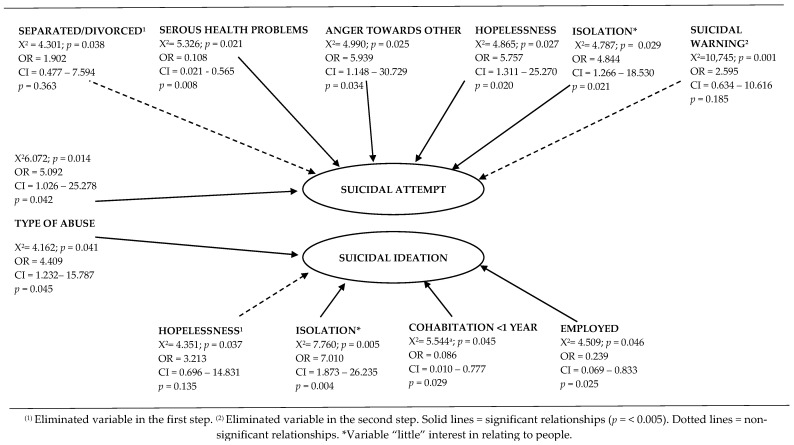
Logistic regression analysis of suicide attempts and suicidal ideation in a group of victimized women. ^(a)^ Fisher’s exact test.

Finally, the analysis showed that the type of violence experienced was statistically significantly related to SI and SAs (*p* < 0.005) (Table 3). In addition to the above, the analysis of differences between the means performed using the Mann–Whitney U statistic showed that women who indicated having experienced SI and SAs had higher scores on the Plutchick suicide risk scale (*p* < 0.001). In this regard, the IPVp+ group also had higher scores than the IPVp group on the said risk scale (*p* < 0.005) (Table 4). The regression models based on the data obtained in this sample indicate that IPVp+ correspond to having a greater risk of generating SI than IPVp (OR = 3.302; CI = 1.027–10.619; *p* < 0.005), as well as SAs (OR = 5.092; 1.026–25.278; *p* < 0.005) (Figure 1).

### 3.4. Logistic Regression Examining Suicide Attempts among Victimized Women

This first analysis shows the relationships between each study factor and SAs (Figure 1). In this sense, the coefficients found allowed a valid regression model (*p* < 0.001) to be constructed for SAs (R^2^ Nagelkerke = 0.472) that, additionally, presents an optimal goodness-of-fit measured through the Hosmer and Lemeshow test (0.944). Thus, the classification table improved at each step of the analysis, classifying 70.8% of the positives. This way, the final regression analysis can predict SAs based on the presence of the related variables. On the other hand, the OR and the p-value improved after eliminating the demographic variable “marital status” (separated and/or divorced) from the analysis in the first step, as well as the variable “suicidal communication” in the second step of analysis. Finally, the final adjustment of this regression model revealed that people exposed to IPVp+ have a greater risk of SA than those who suffer from IPVp (OR = 5.092; CI = 1.026–25.278; *p* = 0.042). Likewise, it was found that the feeling of anger towards others (OR = 5.939; CI = 1.148–30.729; *p* = 0.034), hopelessness (OR = 5.757; CI = 1.311–25.270; *p* = 0.020), and little interest in relating to other people (OR = 4.844; CI = 1.266–18.530; *p* = 0.021) seem to increase the risk of SAs. In contrast, having significant health problems seems to reduce the risk of SAs (OR = 0.108; CI = 0.021–0.565; *p* = 0.008).

### 3.5. Logistic Regression Examining Suicide Ideation among Victimized Women

Logistic regression was also conducted to identify the associated variables of suicidal ideation. This analysis involved several steps in which different variables were progressively included and excluded. After some attempts, a valid final regression model was found (*p* < 0.001) that presents a Nagelkerke R^2^ of 0.396 that correctly classifies 81.8% of the cases. The Hosmer and Lemeshow goodness-of-fit test revealed χ^2^ = 5.947 (*p* = 0.429) (Figure 1).

This second regression model indicates that IPVp+ was significantly related to SI (OR = 4.409; CI = 1.232–15.787; *p* < 0.005) and SAs (OR = 5.092, CI = 1.026–25.278, *p* < 0.005). In this way, isolation experienced by victimized women was the consequence of the abuses perpetrated by their aggressors. This fact would increase the risk of SI (OR = 7.010; CI = 1.873–26.235; *p* = 0.004). Additionally, hopelessness does not show a statistically significant relationship with SI (OR = 3.213; CI = 0.696–14.831; *p* = 0.135), so it was eliminated in the first step of the adjustment. Finally, this regression analysis reveals that cohabitation with an aggressive partner for less than a year (OR = 0.086; CI = 0.010–0.777; *p* = 0.029) would reduce the risk of SI. Similarly, according to the data from this sample, victimized women with an active employment status show a lower risk of developing SI (OR = 0.239; CI = 0.069–0.883; *p* = 0.025).

## 4. Discussion

The main idea behind this study was that IPV could generate a series of consequences, especially hopelessness associated with a higher risk of SB.

In this sense, our first hypothesis was confirmed. There is a high prevalence of psychological violence among women in the sample, going in the same direction as studies previously carried out by author, such as [4,8,11,22].

On the other hand, IPV was considered to be closely related to SI and SAs, and IPVp+ would predictably be associated with a greater risk of suffering these types of behaviours. In this sense, it can be affirmed that this sample of female victims of IPV presents a high prevalence of SI, SC, and SAs. Likewise, the results supported the second hypothesis and confirmed the relationship between IPV and SI, and also with SAs. Thus, these findings point in the same direction as previous research [8,15,27,28,46,47]. As such, gender-based violence does not seem to be limited to the presence of a single type of abuse, but rather implies the combination of several, a fact that turns victims into polyvictims who suffer abuse in several modalities at the same time [48,49]. Additionally, the data confirm that women who experience IPVp+ have a greater risk of experiencing suicidal behaviours, especially when it comes to making SAs [20,21]. Apparently, the stress that the combination of various types of abuse among polyvictimized women could be the reason that explains the increase in SB in its different forms, because the combined violence seems to be the one that causes the greatest health problems [8,10,20,21].

Regarding hopelessness, and contrary to expectations, this characteristic was a relatively infrequent factor in this sample of victimised women. Likewise, depressive symptomatology and hopelessness were not associated with any type of violence (IPVp or IPVp+). Both findings suggest (low frequency of hopelessness and the lack of relationship with the type of IPV) could be explained by the satisfied need for support experienced by the victimized women once they arrive at the PAPs. In this regard, several studies have shown that victims of abuse who seek legal advice or psychological help have better mental health and lower levels of depressive symptomatology, including hopelessness [49,50,51,52]. Nonetheless, as expected in the third hypothesis, the results showed that hopelessness is related to SB. With this, the feeling of hopelessness seems to increase the risk of SAs, although on the contrary it does not seem to increase the risk of suffering SI. This partial confirmation of the third hypothesis differs from studies that have linked hopelessness with an increased risk of SAs and SI [8,15,27,28,35,36,37]. This difference could be attributed to the size of the sample, because, as the study sample was expanded during the data collection, the preliminary analyses showed that the associations between hopelessness and both suicidal behaviours became stronger. Finally, no evidence was found to support the relationship between the years of relationship or cohabitation with hopelessness or with some other consequence of abuse. Thus, it is possible that the consequences of abuse appear in the victims at an early stage. A similar finding was indicated by Cuadrado, Martín-Mora, and Fernández [48], who noted that victims become polyvictims, regardless of the frequency with which the abuses were perpetrated. In contrast, the time of cohabitation seems to be relevant regarding the probability of IPV. In this sense, it was found that the highest proportion of women in this study had maintained the relationship with their aggressors for more than 10 years. This result is consistent with studies that point to a greater risk of IPV as the years of relationship with the aggressor increase [4]. Additionally, cohabitation for less than one year reduces the risk of suffering SI. This finding reveals the importance of an early detection of the victims, a key factor that can make a difference in terms of the potential for SB in victimized women.

Continuing with the consequences of abuse, no evidence was found that either type of violence (IPVp or IPVp+) is related to depressive symptoms or major health problems. Nonetheless, having little interest in relating to people seems to be directly connected to both suicidal behaviours, and increases the risk of SI and SAs, which is consistent with the initial proposition. Additionally, the feeling of anger towards others, although it has been related to both suicidal behaviours, increases the risk of SAs, but not of generating SI. This fact is consistent with research that has indicated that the presence of negative emotions (anger, sadness, anxiety, etc.) increases the probability of the victims’ engaging in risky behaviour [53]. Similarly, and under the right conditions, it is possible for these aversive emotions to become the trigger for even more serious risky behaviour such as SB.

Another factor consistently associated with IPV and suicide risk is having significant health problems [1,54,55]. In this study, around a third of the women reported having significant health problems, with no differences observed between IPVp and IPVp+ or between women with or without SI. Nonetheless, contrary to expectations, having significant health problems reduces the risk of SAs. This result can be explained considering the low frequency of health problems that women in this sample present, particularly among women who attempted suicide at some point in their relationship (only 12% of them had suffered from any major health problem). On the other hand, it seems that not all health problems entail a greater risk of suicide, so it would be interesting to delve into this aspect in future research [56]. In this sense, it is possible that in this specific population, women victimized in relationships, the greater perception of social support perceived by those who regularly visit their doctor modifies the effect that health problems have on SB. It is possible that doctors adopt the role of a significant figure who provides these victims with help and information, ultimately contributing to their feeling more cared for and valued [57].

Likewise, another consequence consistently linked to the abuse experienced and SB is sleep problems [23,56,58]. This factor manifested itself in more than three-quarters of the cases, regardless of the type of violence or the presence of SI or SB. Additionally, the vast majority of the sample reported experiencing feelings of uselessness and failure. This fact suggests that these types of feelings could be related to the repeated attempts to get out of the situation of abuse in which the women found themselves before reaching the PAPs. In this sense, at the time of the interviews, the women continued to be in a vulnerable moment and in the process of recovery.

Finally, the demographic factors that some studies have linked to IPV (age, marital status, etc.) [4,8,10] were not associated with suicidal behaviour in the present study. In this sense, being separated or divorced does not appear to be associated with an increased risk of suicidal behaviour. The relief that victimized women feel when they stop cohabitating with their aggressor could, from this point of view, be the fact that it stops the escalation of severity and minimizes the chances that SI ends up becoming suicide.

On the other hand, as several authors suggest, unemployment can be a risk factor for SB [11]. In this sense, the results showed that the active employment status reduces the risk of experiencing SI. This finding fits well with the results of a meta-analysis [54] which indicated an increased risk of SB among unemployed people. Employed women possibly have greater social support that allows them to cope with the stressful situation [59] deriving, in this case, from abuse.

Regarding the experience of the abuse (running away from home, fear for one’s life, etc.), the importance of the family history has been highlighted in several studies, suggesting a greater risk of suffering IPV when having witnessed intimate partner violence in the family [4,5,6,7,8,9,19]. The results showed that women who suffered IPVp+ witnessed IPV in the family more frequently than IPVp victims. Another significant finding related to the abuse experienced was that most of the women feared for their lives at some point in their relationship with their partner, a very frequent fact among IPV victims [4]. Specifically, in this study, fear is more frequent among women who experienced IPVp+ than any other kind of intimate partner violence. Perhaps this emotion, being more related to physical intimidation by the partner, justifies that in this sample it occurs to a lesser extent among women who only suffered psychological violence [3]. Finally, it is worth highlighting a polyvalent factor, alcohol consumption among victimized women. It is a factor that is considered to be a predictor and, at the same time, a consequence of IPV, in addition to it being a risk factor for suicide. The results indicated a minimal incidence compared to studies that relate IPV with higher levels of consumption, thus agreeing with the opinion of primary care medical professionals who, from their position in detecting victimization, have pointed out that alcohol consumption can be a factor that aggravates the situation of abuse, but that it does not precipitate the phenomenon [60].

## 5. Limitations and Strengths

The results must be interpreted in light of certain limitations. First, one of the limitations of this study is the sample size. With that, it is worth mentioning that the women who go to the PAPs seeking help are usually in a situation of high emotional vulnerability, a fact that makes it difficult to access these victims. Due to the consequences derived from the abuses suffered, many women are not able to participate in this study. Therefore, a large sample is difficult to obtain. Second, it is important to consider that the women included in the sample were already receiving psychological treatment at the time of the study. Third, the analysis was conducted based on retrospective self-reports, so it is possible that the data are underestimated given their stigmatizing nature. Fourth, the study is cross-sectional, therefore the temporality and the order of occurrence of certain events could not be established. Nonetheless, the findings of the study provide invaluable ideas that can be applied in specialized care for victimized women, both from a preventive point of view and during an intervention. In this sense, one strength of the study lies in the knowledge of the associated risk factors of SB in victimized women. This finding can be of great interest for clinical practice because the factors identified in the present study could serve as risk indicators for suicidal behaviour in women victims of IPV. Future studies should include following victims over long periods of time to examine the effect that the interventions have on them in preventing suicide deaths. Additionally, it would be beneficial to explore how the different associated factors of suicidal behaviour in the victimized women found in the present study interact with each other.

## 6. Conclusions

This study suggests that the despair generated in women who suffer IPV can generate SB; this fact is more serious when they suffer psychological violence combined with physical and/or sexual violence. Additionally, the results were consistent with the idea that hopelessness, SI, and previous SAs would increase the risk of carrying out SAs than when there have not been previous attempts.

In conclusion, research concerning the consequences of abuse and the risk of suicide depending on the different types of partner violence can provide information of interest for the development of prevention strategies, both for IPV and suicide, especially taking into account the current lack of studies regarding these topics. Additionally, these findings have implications for policy and practice as they flag the associated factors which should be considered in the attention of victimized women in a therapeutic context. In this sense, the constellation of factors identified in the present study could be included in a specific standard program specifically created for gender-based violence victims. Knowledge of the associated factor with a higher risk of suicidal behaviour could improve the efficiency of both screening and intervention programs by selecting subgroups of women with increased risk of SB. Finally, this work could lead to the implementation of activities to promote the creation of mental health research groups catalogued for inclusion in existing regional, national, and international networks [61].

## Figures and Tables

**Table 1:** Descriptive analysis of the sample.

Variables	Analysis ^1^		Analysis ^2^			Analysis ^3^			Analysis ^4^		
	Total (N = 70) %	X^2^ (*p*) IC 95%	NSI (N = 26) %	SI (N = 44) %	X^2^ (*p*) IC 95%	NSA (N = 24) %	SA (N = 16)	X^2^ (*p*) IC 95%	IPVp (N = 22) %	IPVp+ (N = 48) %	X^2^ (*p*) IC 95%
Age		X^2^ = 30.2 (**)			(n.s.)			(n.s.)			(n.s.)
<29 years	17.1		15.4	18.2		17.4	16.7		9.1	20.8	
30–49 years	64.3		73.1	59.1		65.2	62.5		77.3	58.3	
>50 years	18.6		11.5	22.7		17.4	20.8		13.6	20.8	
Marital status		X^2^ = 30.686 (**)			(n.s.)			(n.s.)			(n.s.)
Single	41.4		53.8	34.1		45.7	33.3		50	37.6	
Married	11.4		7.7	13.6		15.2	4.2		22.7	6.3	
Separated/divorced	47.1		34.6	52.3		39.1	62.5	X^2^ = 4.301 (*)	27.3	56.2	
Years of relationships		X^2^ = 23.771 (**)			(n.s.)			(n.s.)			(n.s.)
1–5 years	25.7		26.9	25		23.9	29.2		31.8	22.9	
6–10 years	14.3		11.5	15.9		13.0	16.7		4.5	18.8	
>10 years	60		61.5	59.1		63.0	54.2		63.6	58.3	
Years of cohabitation		X^2^ = 35.943 (**)			(n.s.)			(n.s.)			(n.s.)
<1 year	11.4		23.1	4.5	X^2^ = 5.544 (*)	13.0	8.3		13.6	10.4	
1–5 years	18.6		7.7	25		15.2	25		13.82	18.8	
6–10 years	14.3		11.5	15.9		15.2	12.5		9.1	16.7	
>10 years	55.7		57.7	54.5		56.5	54.2		59.1	54.2	
Current cohabitation		X^2^ = 51.714 (**)			(n.s.)			(n.s.)			(n.s.)
Alone	10		7.7	11.4		10.9	8.3		0	14.6	
Own family	70		61.5	61.4		54.3	16.7		77.3	64.7	
Birth family	20		23.1	18.2		21.7			22.7	18.8	
Education		X^2^ = 10.314 (*)			(n.s.)			(n.s.)			(n.s.)
Primary	24.3		26.9	22.7		19.6	33.3		13.6	29.2	
Secondary	51.4		50	52.3		52.2	50		59.1	47.9	
University	24.3		23.1	25		28.3	16.7		27.3	22.9	
Laboral status		X^2^ = 25.4 (**)			(n.s.)			(n.s.)			(n.s.)
Unemployed	52.9		38.5	61.4		52.2	54.2		59.1	50	
Employed	41.4		57.7	31.8	X^2^ = 4.509 (*)	41.3	41.7		36.4	43.8	
Student	5.7		3.8	6.8		6.5	4.2		4.5	6.3	

Notes: (1) Descriptive analysis of the sample. (2) Intergroup analysis: Non suicidal ideation vs. Suicidal ideation (NSI vs. SI). (3) Intergroup analysis: Non suicidal intention vs. Suicidal intention (NSA vs. SA). (4) Intergroup analysis: IPVp vs. IPVp+. (4) Bivariate analysis: IPVp vs. IPVp+. (*) Signification *p* < 0.005. (**). Signification *p* < 0.001. (n.s.) = non-significant.

**Table 2:** Qualitative analysis of the experience of gender abuse according to the suicidal behaviour and type of abuse.

Variables	Analysis ^1^		Analysis ^2^			Analysis ^3^			Analysis ^4^		
	Total (N = 70) %	*B* (*p*) IC 95%	NSI (N = 26) %	SI (N = 44) %	X^2^ (*p*) IC 95%	NSA (N = 24) %	SA (N = 16)	X^2^ (*p*) IC 95%	IPVp (N = 22) %	IPVp+ (N = 48) %	X^2^ (*p*) IC 95%
Type of abuse		(*)			X^2^ = 4.16 (*)			X^2^ = 6.07 (*)			-
Psychological abuse	31.4		46.2	22.7		41.3	12.5		100	0	-
Psychological + other type of abuse	68.6		53.8	77.3		58.7	87.5		0	100	-
Family history of IPV	41.4	(n.s.)	30.8	47.7	(n.s.)	34.8	54.2	(n.s.)	22.7	50	X^2^ = 4.62 (*)
Previous psychological treatment	30	(*)	30.8	38.6	X^2^ = 4.21 (*)	26.1	37.5	(n.s.)	18.2	35.4	(n.s.)
Danger for life	85.7	(**)	84.6	86.4	(n.s.)	80.4	95.8	(n.s.)	68.2	93.8	X^2^ = 8.05 ^a^ (*)
Police complaints	72.9	(**)	73.1	72.7	(n.s.)	67.4	83.3	(n.s.)	59.1	79.2	(n.s.)
Runaway from home	64.3	(*)	65.4	63.6	(n.s.)	60.9	70.8	(n.s.)	50	70.8	(n.s.)
Serious health problems	30	(*)	34.6	27.3	(n.s.)	39.1	12.5	X^2^ = 5.326 (*)	31.8	29.2	(n.s.)
Alcohol/Drug abuse	2,9	(**)	0	4.5	(n.s.)	2.2	4.2	(n.s.)	4.5	2.1	(n.s.)

Notes: (1) Descriptive analysis of the sample (Bernoulli binomial distribution). (2) Intergroup analysis: Non suicidal ideation vs. Suicidal ideation (NSI vs. SI). (3) Intergroup analysis: Non suicidal intention vs. Suicidal intention (NSA vs. SA). (4) Intergroup analysis: IPVp vs. IPVp+. (*) Signification *p* < 0.005. (**). Signification *p* < 0.001. (n.s.) = non-significant. ^(a)^ Fisher’s exact test.

**Table 3:** Consequences of gender abuse according to suicidal behaviour and types of abuse.

Variables	Analysis ^1^		Analysis ^2^			Analysis ^3^			Analysis ^4^		
	Total (N = 70) %	X^2^ *(p)* IC 95%	NSI (N = 26) %	SI (N = 44) %	X^2^ *(p)* IC 95%	NSA (N = 24) %	SA (N = 16)	X^2^ *(p)* IC 95%	IPVp (N = 22) %	IPVp+ (N = 48) %	X^2^ *(p)* IC 95%
Psychological treatment	85.7	(**)	88.5	84.1	(n.s.)	84.4	87.5	(n.s.)	81.8	87.5	(n.s.)
Sleep medication	44.3	(n.s.)	34.6	50	(n.s.)	41.3	50	(n.s.)	31.8	50	(n.s.)
Sleep problems	80	(**)	80.8	79.5	(n.s.)	84.8	70.8	(n.s.)	90.9	75	(n.s.)
Health problems	30	(*)	34.6	27.3	(n.s.)	39.1	12.5	X^2^ = 5.326 (*)	31.8	29.2	(n.s.)
Loss of control	54.3	(n.s.)	42.3	61.4	(n.s.)	50	62.5	(n.s.)	45.5	58.3	(n.s.)
Little interest for people	48.6	(n.s.)	26.9	61.4	X^2^ = 7.760 (*)	39.1	66.7	X^2^ = 6.072 (*)	45.5	50	(n.s.)
Pessimistic attitude	51.4	(n.s.)	46.2	54.5	(n.s.)	43.5	66.7	(n.s.)	50	52.1	(n.s.)
Feelings of uselessness	91.4	(**)	84.6	95.5	(n.s.)	91.3	91.7	(n.s.)	90.9	91.7	(n.s.)
Hopelessness	25.7	(**)	11.5	34.1	X^2^ = 4.351 (*)	17.4	41.7	X^2^ = 4.865 (*)	22.7	27.1	(n.s.)
Failure feelings	90	(**)	80.8	95.5	(n.s.)	84.8	100	(n.s.)	81.8	93.8	(n.s.)
Depression feelings	47.1	(n.s.)	42.3	50	(n.s.)	50	41.7	(n.s.)	40.9	50	(n.s.)
Family history of suicide	25.7	(**)	23.1	27.3	(n.s.)	21.7	33.3	(n.s.)	18.2	29.2	(n.s.)
Anger toward others	15.7	(**)	0	25	X^2^ = 7.712 ^a^ (*)	8.7	29.2	X^2^ = 4.990 ^a^ (*)	4.5	20.8	(n.s.)
Suicidal ideation	62.9	(*)	0	100	-	43.5	100	X^2^ = 21.581 (**)	45.5	70.8	X^2^ = 4.162 (*)
Suicidal warning	32.9	(*)	0	52.3	X^2^ = 20.242 (**)	19.6	58.3	X^2^ = 10.745 (*)	18.2	39.6	(n.s.)
Suicidal attempt	34.3	(*)	0	54.5	X^2^ = 21.581 (**)	0	100	-	13.6	43.8	X^2^ = 6.072 (*)
Cur-off point (6)	72.9	(**)	50	95.5	X^2^ = 19.521 (**)	67.4	100	X^2^ = 9.749 (*)	63.6	85.4	(n.s.)

Notes: (1) Descriptive analysis of the sample. (2) Intergroup analysis: Non suicidal ideation vs. Suicidal ideation (NSI vs. SI). (3) Intergroup analysis: Non suicidal intention vs. Suicidal intention (NSA vs. SA). (4) Intergroup analysis: IPVp vs. IPVp+. (*) Signification *p* = < 0.005. (**). Signification *p* = < 0.001. ^(a)^ Fisher’s exact test.

**Table 4:** Quantitative analysis of the experience of gender violence according to suicidal behaviour and type of abuse.

Variables	Analysis ^1^	Analysis ^2^			Analysis ^3^			Analysis ^4^		
	Total (N = 70) (dt)	NSI (N = 26) (dt)	SI (N = 44) (dt)	Mann–Whitney U *(p)*	NSA (N = 24) (dt)	SA (N = 46) (dt)	Mann–Whitney U *(p)*	IPVp (N = 22) (dt)	IPVp + (N = 48) (dt)	Mann–Whitney U *(p)*
Years of relationships with the aggressor	14.2 (10.01)	13.8 (9.81)	14.4(10.24)	(n.s.)	14.8 (10.9)	12.8 (9.1)	(n.s.)	14.6 (10.3)	14 (10)	(n.s.)
Years of cohabitation with the aggressor	11.5 (9.69)	10.9 (10.26)	11.8 (9.45)	(n.s.)	12.3 (10.2)	9.9 (8.6)	(n.s.)	12 (10.3)	11.3 (9.5)	(n.s.)
Suicidal risk	7.77 (2.66)	5.5 (1.70)	9.1 (2.2)	1034 (**)	6.7 (2.2)	9.9 (2.2)	1231 (**)	6.5 (2.1)	8.3 (2.7)	1909 (*)

Notes: (1) Descriptive analysis of the sample. (2) Intergroup analysis: Non suicidal ideation vs. Suicidal ideation (NSI vs. SI). (3) Intergroup analysis: Non suicidal intention vs. Suicidal intention (NSA vs. SA). (4) Intergroup analysis: IPVp vs. IPVp+. (*) Signification *p* < 0.005. (**). Signification *p* < 0.001. (n.s.) = non-significant. (dt) = standard deviation.

## Data Availability

Data are contained within the article.
